# Increasing transparency of computer-aided detection impairs decision-making in visual search

**DOI:** 10.3758/s13423-024-02601-5

**Published:** 2024-10-24

**Authors:** Melina A. Kunar, Giovanni Montana, Derrick G. Watson

**Affiliations:** 1 Department of Psychology, The University of Warwick, Coventry, CV4 7AL UK; 2 Department of Statistics, The University of Warwick, Coventry, CV4 7AL UK

**Keywords:** Artificial intelligence, Computer-aided detection (CAD), Transparency, Low prevalence, Overreliance, Visual search

## Abstract

Recent developments in artificial intelligence (AI) have led to changes in healthcare. Government and regulatory bodies have advocated the need for transparency in AI systems with recommendations to provide users with more details about AI accuracy and how AI systems work. However, increased transparency could lead to negative outcomes if humans become overreliant on the technology. This study investigated how changes in AI transparency affected human decision-making in a medical-screening visual search task. Transparency was manipulated by either giving or withholding knowledge about the accuracy of an ‘AI system’. We tested performance in seven simulated lab mammography tasks, in which observers searched for a cancer which could be correctly or incorrectly flagged by computer-aided detection (CAD) ‘AI prompts’. Across tasks, the CAD systems varied in accuracy. In the ‘transparent’ condition, participants were told the accuracy of the CAD system, in the ‘not transparent’ condition, they were not. The results showed that increasing CAD transparency impaired task performance, producing an increase in false alarms, decreased sensitivity, an increase in recall rate, and a decrease in positive predictive value. Along with increasing investment in AI, this research shows that it is important to investigate how transparency of AI systems affect human decision-making. Increased transparency may lead to overtrust in AI systems, which can impact clinical outcomes.

## Introduction

In recent years, developments in artificial intelligence (AI) have led to important changes in healthcare (Kerasidou et al., 2022). It has been proposed that AI may help with tasks such as workflow and clinical administration (Mello-Thoms & Mello, 2023), the optimisation of clinical trials (Askin et al., 2023), and medical imaging with predictions that AI use in this area will grow dramatically in future years (Allen et al., 2021).

In medical screening, computer-aided detection (CAD) uses computer algorithms to alert readers to the presence of suspicious entities, such as cancers in mammograms. Historically, there have been conflicting results on the benefit of this technology. Although CAD was approved by the Food and Drugs Administration (FDA) in the late 1990s and rolled out at great financial cost (estimated at more than $400 million per annum; Lehman et al., 2015) little research was conducted to examine how these automatic aids affected human decision-making. Subsequent studies showed mixed benefits in relation to CAD use—some positive with increased detection of early-stage malignancies (Freer & Ulissey, 2001), whereas other studies showed little benefit (Lehman et al., 2015). Furthermore, some studies showed harmful results, where CAD led to reduced accuracy of mammogram interpretation (Fenton et al., 2007) and clinicians missed more cancers if not marked by CAD (Taplin et al., 2006; Zheng et al., 2004).

Recent advancements in AI, however, have shown promising results in CAD use. AI acting as a supporting reader produced similar performance to double reading procedures (where two human readers read the same mammogram) while also reducing the number of cases that humans had to read (McKinney et al., 2020; Ng et al., 2023). Given that there is a shortage of available healthcare workers (e.g., Konstantinidis, 2023), the use of AI as a second reader in tasks such as mammography could provide significant benefits.

Despite the potential advantages of AI use in medical screening, there are also disadvantages. Although, the FDA has already approved several AI systems (Benjamens, et al., 2020), the clinical and cognitive costs of human interaction with these systems are still not fully understood and human–AI interactions remain largely underresearched. Furthermore, there is considerable evidence showing that people can become overreliant on this technology (e.g., Buçinca et al., 2021; Bussone et al., 2015; Jacobs et al., 2021; Kunar et al., 2017). This is particularly problematic when the CAD systems either fail to flag a cancer or incorrectly predict the presence of a cancer when there is not one (Kunar et al., 2017). In the former case, cancers that are not prompted by CAD, are more likely to go unnoticed, meaning that women will not receive appropriate and timely medical care. The latter means that women will be needlessly recalled for further tests, which can be worrying and also add an extra and unnecessary burden to healthcare systems (Aro, 2000). Other work has found ways to keep the benefits of CAD while mitigating the costs—for example, by changing the way AI prompts are presented to readers (Kunar, 2022; Patterson & Kunar, 2024) or how the CAD systems are framed (Kunar & Watson, 2023). Crucially, these studies have shown that what people know about AI systems affect how they are used and their influence on decision-making. Given that there is growing investment and support to integrate AI into medical screening (Alexander et al., 2020), it is critical that we examine how such systems influence human decision-making.

With this growth in AI development, government and regulatory bodies have stipulated the importance of transparency within AI systems (Kerasidou et al., 2022; Kingsman et al., 2022; UK Department for Science, Innovation & Technology, 2023). AI transparency can refer to making sure humans are aware of how AI systems operate, and clarity in their accuracy. Transparency can be achieved through various ways. Kiseleva et al. (2022) suggested that factors such as information about an AI system and its interpretability can affect transparency. Lekadir et al. (2021) proposed a set of guidelines for AI use in medical screening, which include that readers should be informed about the errors that AI systems make (e.g., by showing uncertainty estimates) to increase user trust and usability. However, increasing transparency in AI is often complex and can sometimes lead to negative outcomes if people become too dependent on the system (Buçinca et al., 2021). If increased transparency leads to overtrust in the technology, this could have significant consequences for clinical outcomes. For example, increasing transparency about CAD may lead to clinicians accepting the CAD recommendation even if it disagrees with their initial judgement.

We investigated whether increasing transparency of CAD affects search performance in a simulated mammogram task where participants searched for a cancer using seven different CAD conditions. Previous work has shown that laboratory experiments are a reliable way to investigate human reliance on CAD systems (see Kunar et al., 2017, for full details). For example, lab testing allows use of designs that are often not practical, or ethical, in clinical settings, due to the shortage of radiologists and the expense of randomised control trials (RCTs). Furthermore, lab studies allow measures such as miss errors to be observed, which are difficult to determine in a clinical setting given that, by definition, radiologists will be unaware that they have missed a cancer. We can use this measure, along with false alarms and other breast screening metrics such as recall rate (the percentage of mammograms that were reported to have abnormal findings) and positive predictive value (PPV; the percentage of women recalled for further tests who have cancer) to investigate the effects of transparency on decision-making with CAD.

We manipulated transparency across a range of CAD systems that differed in their accuracy to predict a cancer.^1^ CAD transparency was manipulated by explicitly telling people the accuracy of the CAD system before use. In Conditions 1–3, participants were asked to interact with a nontransparent CAD system which either accurately predicted the target on 33% of trials, 67% of trials or 83% of trials. In these conditions participants were not told the accuracy of the CAD algorithm. In Conditions 4–6, participants were shown these same systems but explicitly told in advance the CAD accuracy to make performance more transparent. A final seventh condition acted as a no CAD control, to allow comparison with a baseline where CAD was never used. To preview the results, although making the systems more transparent did not affect miss errors, more transparent systems led to a decrease in performance in relation to false alarms, sensitivity (as measured by *d′*), recall rate, and PPV.

## General method

### Transparency and openness

The data and materials are available on the Open Science Framework (https://osf.io/zrafu/). All data were compiled in Microsoft Excel for Microsoft 365 MSO (Version 2112, Build 16.0.14729.20254) and imported into JASP (Version 0.16; JASP Team, 2021) for statistical analysis. The experimental programs were written in PsychoPy (Peirce et al., 2019) and run via Pavlovia. The study design, hypotheses and analytic plan were not preregistered. All manipulations, data exclusions, and measures are reported.

### Participants

Six-hundred and forty-five participants were tested, in which 100 participants took part in Conditions 1–6 and 44 participants took part in Condition 7.^2^ A G*Power calculation determined that this number of participants resulted in an experimental power above of 0.95, (the minimum number of participants needed per condition to achieve this power was 42, *F* tests, fixed effects, and interactions, alpha = 0.05, effect size = 0.25). Participants were aged 18 or above, recruited via Prolific, and were only able to take part in one experiment. Ethical approval for all studies was granted by the Humanities and Social Sciences Research Ethics Committee at the University of Warwick.

### Stimuli and procedure

Seven conditions were used to examine performance with different CAD systems. The conditions varied by CAD accuracy and whether people were informed of this. In all conditions, participants were asked to search and respond to a mass presented on a mammogram. Two hundred mammogram images were obtained from the Digital Database for Screening Mammography (DDSM; Heath et al., 2001). All original images were randomly selected from the image group that had been confirmed to be cancer free. One hundred and eighty-eight of these images made up the target absent trials (where there was no cancer). The other 12 mammogram images were edited so that they contained a ‘cancer’. To do this, four cancers were chosen at random from cancer cases on the DDSM. For each ‘target-present’ image, one of these cancers was transposed onto a mammogram that previously contained no cancer. Across the experiment all four cancers were transposed onto the mammograms equally often with the premise that only one cancer would appear on each mammogram. Four cancers were chosen so that there would be a degree of perceptual variability across trials increasing the complexity of search, but the target would remain identifiable by medically naïve readers. The mass could appear on any area of the breast tissue, chosen at random (mimicking conditions in a clinical setting), provided that it was clearly distinguishable once fixated. These stimuli and procedure were chosen as they have previously shown to be successful to use in search tasks with participants who have no formal medical training (e.g., Kunar, 2022; Kunar & Watson, 2023; Kunar et al., 2017, 2021; Patterson & Kunar, 2024). At the beginning of the experiment, participants were given a training session, where they were shown example images of the mass and mammograms and asked to detect a mass in a mammogram. They could only continue to the experiment proper, once they had passed the training phase in which they had to correctly respond to whether a mammogram contained a cancer or not at a level above 70% accuracy. If participants failed the training session, they repeated it until their accuracy was above 70%. If participants did not reach this level after their fourth attempt, they were still allowed to proceed, but their data were removed from analysis. However, all participants successfully completed the training phase by this point.

**Fig. 1:**
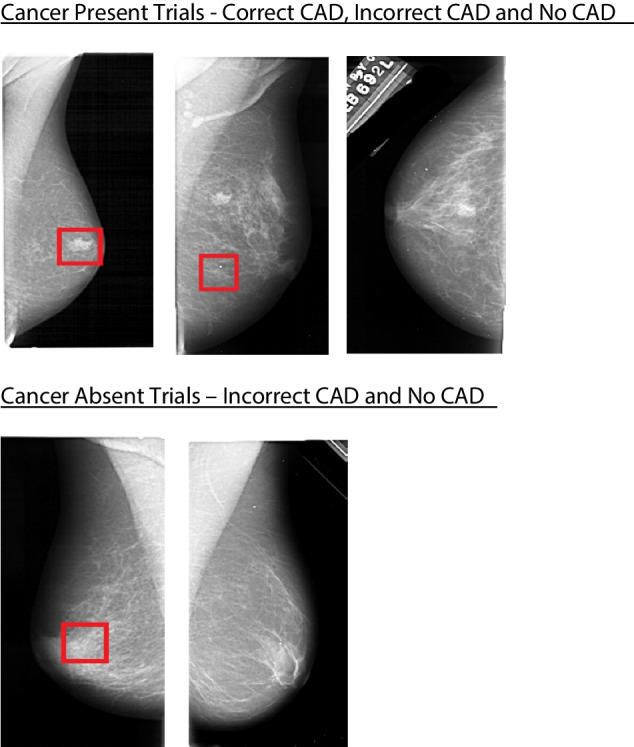
Examples of mammogram displays with correct CAD, incorrect CAD, and no CAD for cancer-present trials and incorrect CAD and no CAD (correct) for cancer-absent conditions

For each condition, the prevalence rate of the cancer was 6%. Conditions 1–6 had participants search for the cancer with the use of a ‘CAD system’ while Condition 7 acted as a baseline where no ‘CAD system’ was used. In Conditions 1–6, participants were informed that they may be shown a CAD prompt in the form of a red box. They were also informed that the CAD cue could be accurate and highlight the target item (correct CAD), but sometimes it could highlight a noncancerous area even when a cancer was present (incorrect CAD) or could contain a cancer which was not flagged by CAD (no CAD). For target-absent trials, there would either be an incorrect CAD cue, which would highlight an area that did not contain a cancer, or no CAD cue would be presented. For target absent trials, the lack of CAD would correctly indicate there was no cancer in the display. Example displays are shown in Fig. 1. Across different experiments, the accuracy of CAD’s ability to correctly highlight the location of the cancer varied, so that it correctly identified a cancer on either 33%, 67%, or 83% of times. Table 1 shows the accuracy rate and number of trials, that either correctly or incorrectly contained a CAD cue, for each condition. All trials were presented in a randomly generated order for each participant. In Conditions 1–3, participants were given no explicit knowledge of the CAD accuracy rate. In Conditions 4–6, participants were explicitly told how accurate the CAD system was (e.g., ‘In this session the CAD cue [red box] will highlight the cancer 83% of the time’). In Condition 7, participants were asked to search for cancers without the use of a ‘CAD system’. In this condition, participants responded to whether a mammogram contained a cancer or not. None of the mammogram images contained a CAD cue, and CAD was not mentioned in the instructions.

**Table 1:** Summary of accuracy rates and trial numbers for each condition

Condition	Overall CAD accuracy rate	Transparent knowledge of CAD accuracy	Absent trials with CAD	Absent trials with no CAD	Present trials with correct CAD	Present trials with incorrect CAD	Present trials with no CAD
1	33%	No	47	141	4	4	4
2	67%	No	47	141	8	2	2
3	83%	No	47	141	10	1	1
4	33%	Yes	47	141	4	4	4
5	67%	Yes	47	141	8	2	2
6	83%	Yes	47	141	10	1	1
7	n/a	n/a	0	188	0	0	12

CAD accuracy refers to accurate detection of a cancer when it is present. Condition 7 acted as a no CAD control. n/a =

For each trial, participants were asked to respond whether there was a cancer in the mammogram image. If they believed there was a cancer, they pressed the ‘m’ key. If they believed there was no cancer, they pressed the ‘z’ key. To ensure that the results were not affected by motor errors (Fleck & Mitroff, 2007), participants had to respond a second time, to confirm their response. This was done by again pressing the ‘m’ key for target-present responses and ‘z’ key for target-absent responses. This ensured that participants could correct their initial response if they accidently pressed the wrong button. Participants were given a short practice session before the start of the experiment.

### Data analysis

Incomplete data sets were removed from analyses. This led to the removal of six participants (one participant in Conditions 1, 3, 6, and 7, two participants in Condition 4). To avoid motor errors, the confirmed responses were used to calculate miss errors and false alarms. If performance was negatively affected by transparency, we would expect to see a greater proportion of miss errors and/or a greater proportion of false alarms in the transparent versus the not transparent CAD conditions.

To understand the reason for any differences in error rates across experiment, we examined how sensitivity (as measured by *d′*) and response bias (measured by *c*) changed across CAD systems using signal-detection theory (SDT; Green & Swets, 1967; Macmillan & Kaplan, 1985). If performance was negatively affected by transparency, we would expect to see a decrease in *d′* in the transparent compared with the not transparent CAD conditions. A change in criteria across transparency would also suggest a shift in response bias, with a higher criteria reflecting that participants were less willing to respond that a cancer was present.

The data were also analysed to see how transparency affected recall rates and PPV. Recall rate and PPV are important clinical metrics within breast-cancer screening (e.g., Norsuddin et al., 2015; Rauscher et al., 2021; Taylor-Phillips et al., 2024) and were calculated as follows, in which TP stands for true positive, FP stands for false positive (false alarms), TN stands for true negative, and FN stands for false negative:

$$Recall Rate= \frac{\sum (TP+FP)}{\sum (TP+FP+TN+FN)} \times 100$$

$$PPV= \frac{\sum TP}{\sum (TP+FP)} \times 100$$

If adding transparency affected performance negatively, we would expect to see higher recall rates and lower PPV in the transparent conditions compared with the not transparent conditions.

For each of these metrics we conducted a 3 × 2 analysis of variance (ANOVA) with between factors of CAD accuracy (33%, 67%, and 83%) and transparency (transparent vs not transparent). Significant interactions were further analysed using planned *t* tests, where we also include Bayesian analyses, as supportive evidence (Wagenmakers et al., 2018a). We only include Bayesian analysis for these planned *t* tests rather than ANOVAs as the latter is still an ongoing topic of research (Wagenmakers et al., 2018b). For our Bayesian analyses, we adopt the recommendations of Jeffreys (1961), in which a BF_10_ of 1 to 3 provides *anecdotal* evidence for the alternative, a BF_10_ of 3 to 10 provides *substantial* evidence for the alternative, a BF_10_ of 10 to 30 provides *strong* evidence for the alternative, a BF_10_ of 30 to 100 provides *very strong* evidence for the alternative and a BF_10_ of greater than 100 provides *decisive* evidence for the alternative. The inverse of these numbers (BF_01_) provide evidence in support the null hypothesis (Jarosz & Wiley, 2014).

Lastly, data from each CAD Condition (1–6) were compared with the no CAD control (Condition 7). This enabled us to determine whether there was an overall benefit or cost of CAD, in relation to when no CAD system was used. For each metric, six *t* tests were run. To compensate for multiple comparisons, we used the Bonferroni correction for with the adjusted alpha levels of 0.008 per test (0.05/6).

## Results

**Fig. 2:**
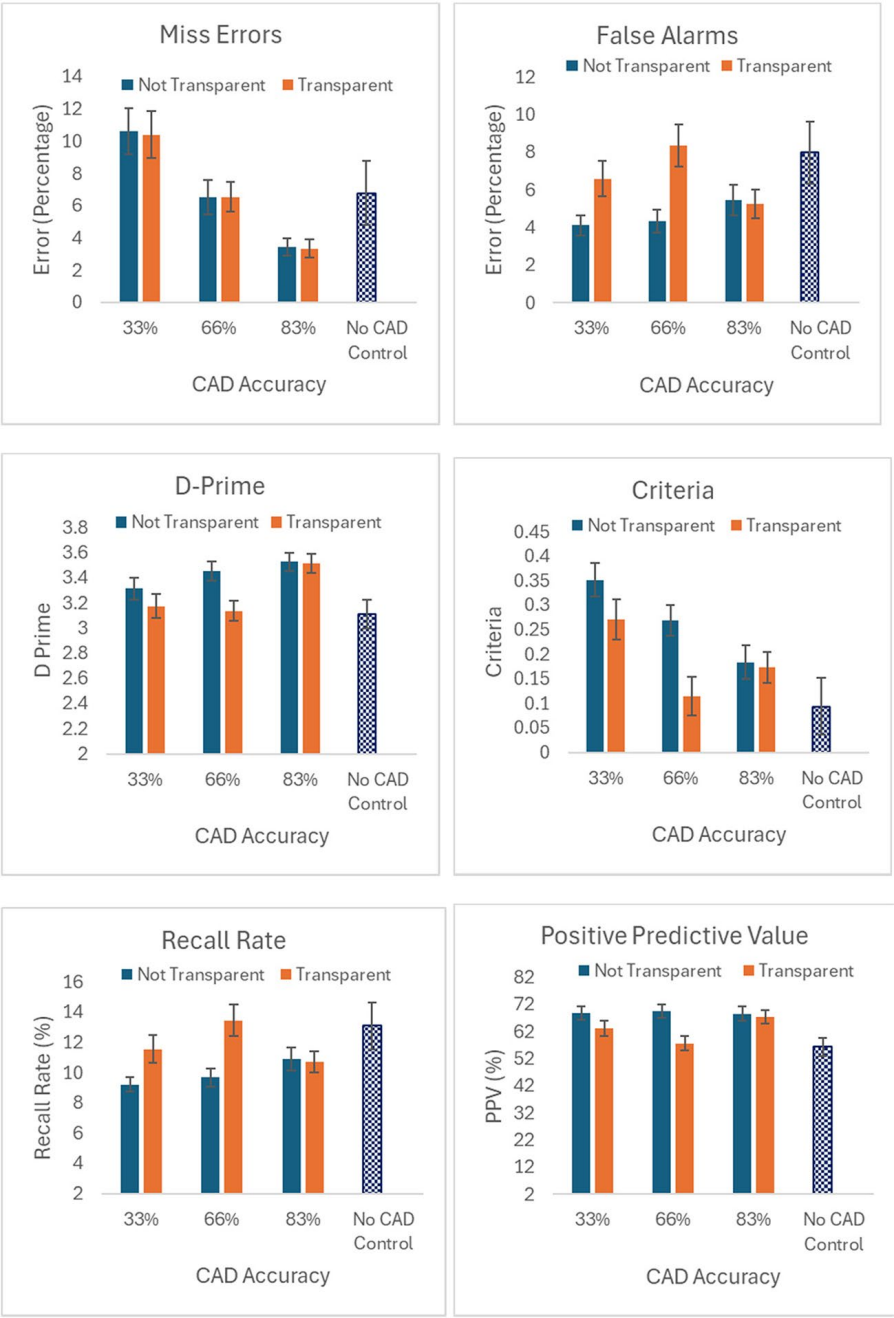
Mean values across conditions. *Note.* Error bars represent the standard error

Figure 2 shows the data for all conditions.

### Miss errors

For miss errors, the 3 × 2 ANOVA revealed a main effect of CAD accuracy, *F*(2,590) = 22.64, *p* < 0.001, η_p_^2^ = 0.07, in which miss errors decreased with increasing CAD accuracy. Neither the main effect of transparency, *F*(1,590) = 0.02, *p* = 0.90, η_p_^2^ < 0.001, nor the CAD Accuracy × Transparency interaction were significant, *F*(2,590) = 0.005, *p* = 0.995, η_p_^2^ < 0.001.

Comparisons of individual conditions with the no CAD control showed that there were no significant differences in miss errors (see Table 2 for details of all comparisons).

**Table 2:** Comparisons of each CAD condition with the no CAD control

Condition compared with the no CAD control	Metric	CAD accuracy rate	Transparent knowledge of CAD accuracy	*t*	*df*	*p*
1	Miss errors	33%	Not transparent	1.57	142	.13
2		67%	Not transparent	0.10	140	.92
3		83%	Not transparent	2.17	140	.03
4		33%	Transparent	1.42	141	.16
5		67%	Transparent	0.12	139	.90
6		83%	Transparent	2.17	140	.03
1	False alarms	33%	Not transparent	2.84	142	.005**
2		67%	Not transparent	2.51	140	.01
3		83%	Not transparent	1.54	140	.13
4		33%	Transparent	0.77	141	.45
5		67%	Transparent	0.19	139	.85
6		83%	Transparent	1.74	140	.09
1	*D* prime	33%	Not transparent	1.35	142	.18
2		67%	Not transparent	2.47	140	.015
3		83%	Not transparent	3.15	140	.002**
4		33%	Transparent	0.39	141	.70
5		67%	Transparent	0.19	139	.85
6		83%	Transparent	3.02	140	.003**
1	Criteria	33%	Not transparent	4.02	142	< .001**
2		67%	Not transparent	2.85	140	.005**
3		83%	Not transparent	1.40	140	.16
4		33%	Transparent	2.45	141	.02
5		67%	Transparent	0.29	139	.77
6		83%	Transparent	1.30	140	.20
1	Recall rate	33%	Not transparent	3.03	142	.003**
2		67%	Not transparent	2.51	140	.01
3		83%	Not transparent	1.41	140	.16
4		33%	Transparent	0.89	141	.37
5		67%	Transparent	0.20	139	.85
6		83%	Transparent	1.60	140	.11
1	PPV	33%	Not transparent	2.86	142	.005**
2		67%	Not transparent	3.07	140	.003**
3		83%	Not transparent	2.66	140	.009
4		33%	Transparent	1.37	141	.17
5		67%	Transparent	0.28	139	.78
6		83%	Transparent	2.45	140	.02

** *p* values are significant using the adjusted Bonferroni correction alpha level of 0.008 per test (.05/6)

### False alarms

For false alarms, the 3 × 2 ANOVA revealed no main effect of CAD accuracy, *F*(2,590) = 0.98, *p* = 0.374, η_p_^2^ = 0.003. There was a main effect of transparency *F*(1,590) = 9.94, *p* = 0.002, η_p_^2^ = 0.017 0.001, with more false alarms in the transparent CAD conditions. The CAD Accuracy × Transparency interaction was also significant, *F*(2,590) = 3.42, *p* = 0.03, η_p_^2^ = 0.01. Planned *t* tests showed that false alarms were higher for transparent CAD systems when the CAD accuracy rate was 33%, *t*(199) = 2.28, *p* = 0.02, *d* = 0.32, with anecdotal evidence in support of the alternative, BF_10_ = 1.70, and when the CAD accuracy was 66%, *t*(195) = 3.17, *p* = 0.002, *d* = 0.45, with strong evidence in support of the alternative, BF_10_ = 15.73. There was no effect of transparency when the CAD accuracy was 83%, *t*(196) = 3.18, *p* = 0.85, *d* = 0.03, with substantial evidence in support of the null, BF_10_ = 0.16.

Comparisons of individual conditions with the no CAD control showed fewer false alarms in the 33% not transparent CAD condition compared with the no CAD control. There were no other significant differences.

### Sensitivity (*d’*)

The 3 × 2 ANOVA revealed a main effect of CAD accuracy, *F*(2,590) = 6.67, *p* = 0.001, η_p_^2^ = 0.02, in which *d′* increased with increased CAD accuracy. There was also a significant main effect of transparency, *F*(1,590) = 5.71, *p* = 0.02, η_p_^2^ = 0.01, in which *d′* was lower in the transparent CAD conditions. The CAD Accuracy × Transparency interaction was not significant, *F*(2,590) = 1.77, *p* = 0.17, η_p_^2^ = 0.006.

Comparisons of individual conditions with the no CAD Control showed that *d′* was higher in both the 83% accuracy not transparent and transparent conditions in comparison with the no CAD control. There were no other significant differences.

### Criterion (*c*)

The 3 × 2 ANOVA revealed a main effect of CAD accuracy, *F*(2,590) = 8.68, *p* < 0.001, η_p_^2^ = 0.03, in which people were more willing to respond that a target was present as CAD accuracy increased. There was also a significant main effect of transparency, *F*(1,590) = 8.07, *p* = 0.005, η_p_^2^ = 0.01, in which people were more willing to respond that a cancer was present in the transparent CAD conditions. The CAD Accuracy × Transparency interaction was not significant, *F*(2,590) = 2.10, *p* = 0.12, η_p_^2^ = 0.007.

Comparisons of individual conditions with the no CAD control showed that participants were more willing to say a cancer was present in the no CAD control compared with the 33% not transparent CAD condition and to the 67% not transparent CAD condition. There were no other significant differences.

### Recall rate

The 3 × 2 ANOVA on recall rate showed no main effect of CAD accuracy, *F*(2,590) = 1.21, *p* = 0.30, η_p_^2^ = 0.0043. There was a significant main effect of transparency, *F*(1,590) = 10.11, *p* = 0.002, η_p_^2^ = 0.02, in which recall rate was higher in the transparent CAD conditions. The CAD Accuracy × Transparency interaction was also significant, *F*(2,590) = 3.45, *p* = 0.03, η_p_^2^ = 0.01. Planned *t* tests showed that recall rate was higher in the transparent CAD conditions when the CAD accuracy was 33%, *t*(199) = 2.31, *p* = 0.02, d = 0.33, with anecdotal evidence in support of the alternative, BF_10_ = 1.8, and when the CAD accuracy was 66%, *t*(195) = 3.18, *p* = 0.002, *d* = 0.45, with strong evidence in support of the alternative, BF_10_ = 16.32. However, there was no reliable difference across Transparency when the CAD accuracy was 83%, *t*(196) = 3.18, *p* = 0.86, *d* = 0.03, with substantial evidence in support of the null, BF_10_ = 0.16.

Comparisons of individual conditions with the No CAD Control showed that the recall rate was lower in the 33% not transparent CAD condition. There were no other significant differences.

### Positive predictive value (PPV)

The 3 × 2 ANOVA on PPV showed no main effect of CAD accuracy, *F*(2,590) = 1.40, *p* = 0.25, η_p_^2^ = 0.005. There was a significant main effect of transparency, *F*(1,590) = 9.11, *p* = 0.003, η_p_^2^ = 0.02, in which PPV was lower in the transparent CAD conditions. The CAD Accuracy × Transparency interaction was not significant, *F*(2,590) = 2.15, *p* = 0.12, η_p_^2^ = 0.007.

Comparisons of individual conditions with the no CAD control showed that the PPV was higher in the 33% not transparent CAD condition and the 67% not transparent CAD condition. There were no other significant differences.

## General discussion

This study examined the effect of transparency on a range of CAD systems that varied in their predictive accuracy. Not surprisingly, the more accurate CAD systems led to better target detection. This increase in accuracy was mostly observed in fewer miss errors given that the CAD accuracy manipulation was specific to target present trials only. More importantly transparent CAD conditions showed an impairment in performance with increased false alarms, decreased sensitivity, a less conservative response threshold, an increase in recall rate (more women being unnecessarily recalled in a clinical setting), and a decrease in PPV (fewer women being recalled who actually had cancer). Overall, the data showed that increasing transparency by informing people about the accuracy of the CAD system led to negative performance across a number of metrics. This is of concern, given recent government and regulatory body recommendations that AI systems should show increased transparency (Kerasidou et al., 2022).

The shift in false alarms, recall rate and PPV can be explained by the SDT data, which indicated that participants showed a decrease in sensitivity in the transparent conditions and were more likely to report a cancer was present. Wolfe and Van Wert (2010) proposed a multiple-decision model (MDM) to account for visual search data based on two factors: (i) the amount of time spent searching an image before concluding a target is not there (the ‘quitting threshold’) and (ii) the amount of evidence above which a target is deemed as present (the response threshold). These factors can be affected by target prevalence (e.g., Wolfe et al., 2007) and by the addition of CAD (e.g., Kunar, 2022). Our data add to this model by showing that CAD transparency also affects a person’s response threshold above which they are willing to accept a target as present.

Comparing performance of the CAD conditions to the no CAD control we see mixed results. Somewhat surprisingly, evidence that performance in the CAD conditions was superior to the no CAD condition was underwhelming. This is particularly true of the transparent CAD conditions, which showed little difference compared to the no CAD control. One exception was that sensitivity in the transparent condition was higher than the no CAD control with a CAD accuracy rate of 83%. However, this did not translate to better performance in the other metrics. In the not transparent conditions, there was some improvement over the no CAD baseline. However, this mostly occurred when the CAD accuracy rate was lower.^3^ Sensitivity was improved in the 83% CAD accuracy condition, but again this did not translate to improvement in other measures. Given that CAD systems in a clinical setting would be expected to show a high degree of accuracy, it is interesting that these systems only showed little improvement in performance compared with no CAD conditions. However, given that other research has shown beneficial effects of CAD over no CAD systems (e.g., Drew et al., 2020), future research would be needed to investigate this further.

One reason why giving people explicit knowledge about CAD accuracy changes their decision outcomes may be because it affects their dependency on those systems. If transparency leads to overtrust in the CAD system, participants would be more likely to accept the CAD recommendation, even if it disagrees with their own judgement (see Felzmann et al., 2020, for a discussion on the link between transparency and trust in AI). Given that explainable AI (XAI) is a complex and difficult field of research (Biran & Cotton, 2017; Scharowski et al., 2023), the above results question whether the need to produce transparent AI is always necessary. Further research needs to be conducted on this, but for present purposes our data clearly show that increased transparency about AI accuracy can lead to negative effects.

Lastly, it may be that participants chose not to use the CAD system if they were informed of its inaccuracy. There is some evidence that this may be the case if we examine Fig. 2, which shows differences in metrics at the lower CAD accuracy rates (33% and 67%) across transparent and not transparent conditions. This may suggest that when participants were informed of a lower accuracy rate in the transparent conditions that the CAD systems were being *underused.*^4^ Given that we did not measure people’s perceptions of the CAD systems in these experiments, we are unable to determine this here. Nevertheless, this is an important avenue of research for future work.

The current results are important for medical screening in clinical settings. However, there are differences between lab-based studies and medical screening which need further exploration. For example, the prevalence rate of breast cancer would be lower in the clinical setting, and radiologists, of course, have greater expertise than our participants. Furthermore, we operationalised transparency as the level of explicit knowledge available about a system’s accuracy. However, there are other factors that affect transparency (Kiseleva et al., 2022). Future research is needed to examine these factors further. However, for now, the data suggest that the regulatory goal of making AI systems more transparent may not always lead to positive outcomes.

## Data Availability

The data and materials for all experiments are available online (https://osf.io/zrafu/).

## Footnotes

1 In a clinical setting the accuracy of CAD is thought to be in the range of 57%–85% but this can vary across systems (Soo et al., 2005; Obenauer et al., 2006). In these experiments a CAD accuracy range of 33% to 83% was used to test whether there were differential effects of transparency on CAD systems that had low or high accuracy rates.

2 A total of 101 participants took part in Condition 2 due to an error with Prolific—for ethical reasons we chose to analyse all data sets collected.

3 With decreased false alarms, lower recall rate, and increased PPV in the 33% accuracy condition, and increased PPV in the 67% accuracy condition.

4 We thank Todd Horowitz for this suggestion.

## Declarations

### Open practices statement

The data and materials for all experiments are available online (https://osf.io/zrafu/). None of the experiments were preregistered.
